# A survey of practice patterns for adaptive particle therapy for interfractional changes

**DOI:** 10.1016/j.phro.2023.100442

**Published:** 2023-04-28

**Authors:** Petra Trnkova, Ye Zhang, Toshiyuki Toshito, Ben Heijmen, Christian Richter, Marianne C. Aznar, Francesca Albertini, Alessandra Bolsi, Juliane Daartz, Antje C. Knopf, Jenny Bertholet

**Affiliations:** a Department of Radiation Oncology, Medical University of Vienna, Vienna, Austria; b Center for Proton Therapy, Paul Scherrer Institute, Villigen, Switzerland; c Nagoya Proton Therapy Center, Nagoya City University West Medical Center, Nagoya, Japan; d Department of Radiotherapy, Erasmus University Medical Center (Erasmus MC), Rotterdam, the Netherlands; e OncoRay – National Center for Radiation Research in Oncology, Faculty of Medicine and University Hospital Carl Gustav Carus, Technische Universität Dresden, Helmholtz-Zentrum Dresden – Rossendorf, Dresden, Germany; f Division of Cancer Sciences, Faculty of Biology, Medicine and Health, University of Manchester, Manchester, United Kingdom; g Department of Radiation Oncology, Massachusetts General Hospital & Harvard Medical School, Boston, MA 02114, United States of America; h Institute for Medical Engineering and Medical Informatics, School of Life Science FHNW, Muttenz, Switzerland; i Division of Medical Radiation Physics and Department of Radiation Oncology, Inselspital, Bern University Hospital, Bern, Switzerland

**Keywords:** Particle/proton therapy, Adaptive radiotherapy (ART), Interfraction anatomical variation, Image guided particle therapy, Adaptive treatment planning

## Abstract

**Background and purpose:**

Anatomical changes may compromise the planned target coverage and organs-at-risk dose in particle therapy. This study reports on the practice patterns for adaptive particle therapy (APT) to evaluate current clinical practice and wishes and barriers to further implementation.

**Materials and methods:**

An institutional questionnaire was distributed to PT centres worldwide (7/2020–6/2021) asking which type of APT was used, details of the workflow, and what the wishes and barriers to implementation were. Seventy centres from 17 countries participated. A three-round Delphi consensus analysis (10/2022) among the authors followed to define recommendations on required actions and future vision.

**Results:**

Out of the 68 clinically operational centres, 84% were users of APT for at least one treatment site with head and neck being most common. APT was mostly performed offline with only two online APT users (plan-library). No centre used online daily re-planning. Daily 3D imaging was used for APT by 19% of users. Sixty-eight percent of users had plans to increase their use or change their technique for APT. The main barrier was “lack of integrated and efficient workflows”. Automation and speed, reliable dose deformation for dose accumulation and higher quality of in-room volumetric imaging were identified as the most urgent task for clinical implementation of online daily APT.

**Conclusion:**

Offline APT was implemented by the majority of PT centres. Joint efforts between industry research and clinics are needed to translate innovations into efficient and clinically feasible workflows for broad-scale implementation of online APT.

## Highlights

• 84% of particle therapy centres adapt plans for inter-fractional changes.
• All users of adaptive particle therapy except two perform offline plan adaptation.
• 68% of centres had plans to implement or improve their adaptive workflow.
• The lack of integrated workflows was the main limitation to implementation.
• Broad-scale implementation needs joint efforts of industry, research and clinics.

## 1 Introduction

Particle therapy (PT) is sensitive to interfractional anatomical changes potentially leading to significant dose difference between the planned and delivered dose [1], [2], [3]. Geometrical population-based margins [4] or robust optimization [5] account for positioning and range uncertainties. It may, however, be insufficient for large changes caused by tumour shrinkage, weight-loss, and organ size and shape variations. Moreover, these approaches impose increased dose to organs-at-risk (OARs) [6]. During adaptive PT (APT) more than one treatment plan per target per course is created to mitigate the detrimental effect of anatomical changes, potentially improving target coverage and OAR sparing [7], [8].

Adaptation can be performed either offline or online [9], [10]. Offline adaptation, requiring often up to several days for implementation of a new treatment plan, is adequate for slow progressive changes. Adaptation can be triggered ad-hoc when observing large changes, or per protocol with regular evaluation of (predefined) geometric or dose parameters and/or scheduled surveillance scans [11]. Another approach used for large but predictable changes (e.g. different levels of organ filling) is plan-library, where a set of plans is created for several anatomical situations and the most adequate plan is selected online at each fraction [12], [13].

Daily re-planning can potentially address any type of interfractional variation based on imaging-of-the-day when performed within minutes [14]. There are several strategies such as full re-planning [15], [16], dose restoration [17], [18] or machine learning based dose prediction [19]. In photon therapy, daily re-planning has become more common in recent years [20], [21], [22], [23], [24], [25], [26]. The status of clinical implementation of daily re-planning in PT is unknown but extensive research is ongoing [14].

With the aim to identify the status of APT clinical implementation, plans for improvements in individual PT centres and barriers to clinical implementation, a worldwide survey addressing practice patterns was initiated. Although the challenges of plan adaptation are similar to photon therapy, the clinical need may be higher for PT because of the dosimetric properties of the applied beams. Furthermore, the technology (and commercial availability thereof) for image-guidance and plan adaptation for PT differs widely [3]. The survey was followed by a Delphi consensus analysis among the authors to provide recommendations for the particle therapy community on the next steps in research, development and clinical implementation.

## 2 Material and methods

### 2.1 Survey

The web-based Patterns Of Practice for Adaptive and Real-Time motion management for particle therapy (POP-ART PT) questionnaire was adapted from a survey for photon therapy [27], [28], validated by the co-authors and endorsed by European Society for Radiotherapy and Oncology (ESTRO), European Particle Network (EPTN) and Particle Therapy Co-Operative Group (PTCOG). It targeted clinical medical physicists and focused on current clinical practice at the institutional level and wishes and barriers to implementation. Data were collected between 7/2020 and 6/2021. Centres listed on the PTCOG website as Facilities in Operation (https://www.ptcog.ch) in 2020 were invited to participate using mailing list, social media and personal contacts.

This paper focuses on APT for interfractional anatomical changes. Real-time respiratory motion management is addressed in a parallel paper [29]. The following APT strategies were considered in the survey: offline ad-hoc adaptation due to e.g. occasional detection of large changes such as tumour shrinkage or weight loss; offline re-planning per protocol with pre-defined objective measures (e.g. geometric change above a certain threshold) based on either image-of-the-day or scheduled surveillance scans (e.g. at given time-point in the treatment course) to decide on the need for a new treatment plan; online with a plan-library; online daily re-planning. Supplementary material (Suppl. A) contains all the questions.

Responding centres (“responders” thereafter) were PT centres who provided a complete response or left only a few questions unanswered (marked as ‘‘not specified”). Multiple answers from a single institution were merged and checked for consistency. In case of inconsistent answers, the responders were contacted for clarification. The responders had also the opportunity to provide free-form comments to some questions (marked “free comment”).

Seventy PT institutions from 17 countries worldwide completed the questionnaire (details in Suppl. B). The response rate was 100% for Europe (23/23), 96% for Japan (22/23) and 53% for USA (20/38). Additionally, four centres from China and one from Thailand participated. Two responders had not yet started clinical application but filled the wish-list questions.

### 2.2 Delphi consensus analysis

To formulate recommendations on required actions and future vision, a process inspired by the Delphi consensus analysis [30] was performed among all authors. A concept questionnaire was developed by four authors (PT, YZ, AK, JB) and revised by the rest. All authors answered the questions and sent them to the moderator of the analysis (PT). The moderator summarised the answers and redistributed the questionnaire with answers in an anonymized form. Authors could revise their answers and comment on the answers from others. The moderator participated in answering the questionnaire, however, always prior to reading the answers from the others.

The Delphi consensus analysis was performed in three rounds. Experts were asked to answer based on their interpretation of the survey results addressing wish-list and barriers as well as their personal opinion and experience. Full consensus (FC) or partial consensus (PC) were reached when all experts agreed or only one expert had a different opinion, respectively. The details on the Delphi analysis including its organisation and questions of the third round are provided in Suppl. C.

## 3 Results

Out of all the clinically operational responders (“clinical responders” hereafter, N = 68), 84% (57/68) were APT users with 72% (41/57) using ad-hoc offline APT, 58% (33/57) using APT per protocol and 32% (18/57) using both offline APTs for at least one treatment site (Table 1). One Japanese centre performed online plan adaptation with plan-library for all their APT indications, however, for some indications, offline APT per protocol was used as an alternative. One US-centre indicated using a plan-library with alternative ad-hoc re-planning, however, without specifying the treatment site. The most common treatment site for APT was head and neck where APT was applied by 95% (54/57) of the users. More details are provided in Suppl. B (Fig. B.1-2, Tables B.1–4). In addition to the predefined treatment sites, 19 “other” treatment sites (free comment) were named (Suppl. Table B.5).

**Table 1:** An overview of the number of users per indication out of 68 clinical responders.

Treatment site	APT users	Type of adaptation					
		OFFLINE			ONLINE		not specified
		ad-hoc	per protocol	both	Plan-library	Daily replanning	
***Any site***	***57 (84%)***	***41***	***33***	***18***	2*°	***0***	***1***
Bladder	11 (16%)	10	2	*1*	0	0	0
Cervix	12 (18%)	7	7	*2*	1*	0	0
Rectum	21 (31%)	16	8	*4*	1	0	0
Prostate	28 (41%)	21	11	*6*	1	0	1
Head and neck	54 (79%)	35	30	*12*	1*	0	1
Lung	42 (62%)	27	19	*6*	1*	0	2
Other	40 (59%)	25	22	*9*	2*°	0	2

*One centre performed offline APT per protocol and online library of plans.

°One centre performed offline ad-hoc APT and online library of plans.

**Fig. 1:**
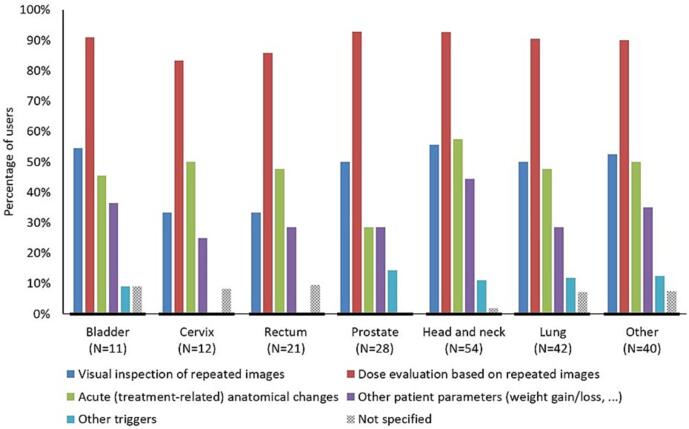
An overview of the information that lead to plan adaptation. Each user could provide multiple answers. The percentage of users is related to the number of users per treatment site (N).

The treatment site with the least frequent adaptation was prostate where most users reported creating more than one plan for less than 5% of the patients (Suppl. Fig. B.3). Head and neck had the most frequent adaptations with 63% of users adapting for more than 25% of their patients. For all users, plan adaptation was motivated by both target coverage and OAR dose. The main trigger was dose evaluation on repeated images used by 90% or more users for all treatment sites, often together with other criteria (Fig. 1). Triggers reported as “other” were (free-comment): positioning or immobilization device problems, log-file recalculation of the previous fraction, deep-inspiration breath-hold instability, fluctuating saline injection quantity for bladder cancer, insertion or exchange of a stent for bile duct or pancreatic cancer, shifted breast implant, and ad-hoc doctor’s decision.

Most often, a combination of different imaging modalities was used during APT, e.g., 2D X-ray imaging or cone-beam computed tomography was used as a first trigger to decide on the acquisition of additional planning computed tomography (CT) or magnet resonance imaging (MRI), which was afterwards used for dose recalculation and final decision on plan adaptation. Additional CT was used to generate the adapted plan by 64–78% of the users depending on the treatment site. Only 19% (11/57) of the users performed daily 3D APT imaging. For offline APT, online imaging was used to trigger adaptation but not to create the adapted plan. More details can be found in Suppl. B (Figs. B.4–6).

**Fig. 2:**
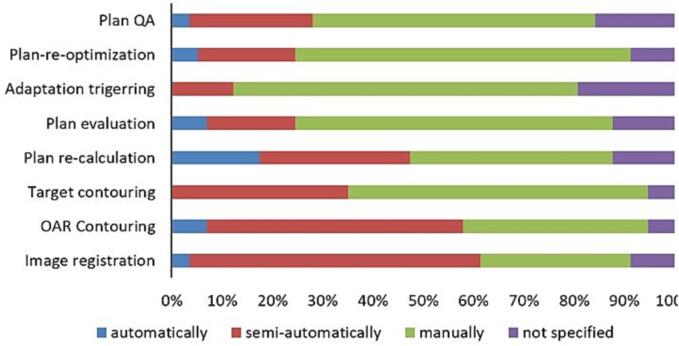
An overview of the level of automation in individual workflow step over N = 57 APT users.

In-house developed software was employed for at least one step of the APT workflow by 18% of users (10/57): eight for plan quality assurance (QA), two each for image registration, target contouring, plan dose re-calculation, plan evaluation, plan re-optimization and one for OAR contouring. One centre used open-source software for plan dose re-calculation. In all other cases, commercially available software was used. The main reason to use in-house developed software was “insufficient functionality of the commercially available software” (N = 7), followed by “unavailability of the commercial solution” (N = 3), “lack of connectivity” (N = 3), “unfinished commissioning of the software” (N = 3), “not good enough solutions” (N = 3) and “costs of commercial software” (N = 3). All the steps of the APT workflow were performed mainly manually (Fig. 2). Plan recalculation was automatized by 18% of the users (10/57). None of the users automatically derived target contours or triggers for adaptation.

**Fig. 3:**
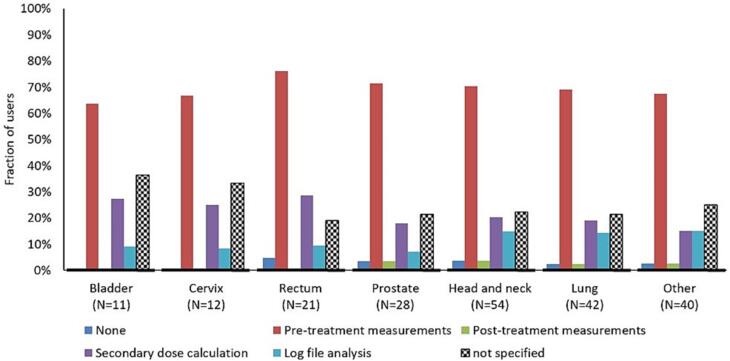
The type of QA performed for APT plans. Multiple answers were possible. The percentage of users is related to the number of APT users per treatment site (N).

For all users except one, the QA procedure was the same for the adapted plan as for the original plan. The most common QA method was pre-treatment phantom measurements followed by secondary dose calculation (Fig. 3). Thirty percent (17/57) of the users were performing more than one QA type for the APT plans. All users, except one, were documenting the impact of plan adaptations. The most used system was record and verify (60%) followed by treatment planning system (30%) and spreadsheet (10%).

Sixty-eight percent of users (39/57) had plans to increase the use of APT or change their workflow. The details on the intended changes in APT are provided in Suppl. B (Figs. B.7–8). One centre explicitly stated that they would like to introduce an online adaptive workflow (free comment). Other centres did not specify what kind of improvements they would like to introduce. Sixty percent of responders (42/70) wanted to introduce APT for a new treatment site and scored barriers to implementation. From the non-users, 69% (9/13) would like to introduce APT in the clinic however they did not state which treatment site was a priority. For users (N = 57), liver and lung were most commonly stated as new future APT treatment site.

**Fig. 4:**
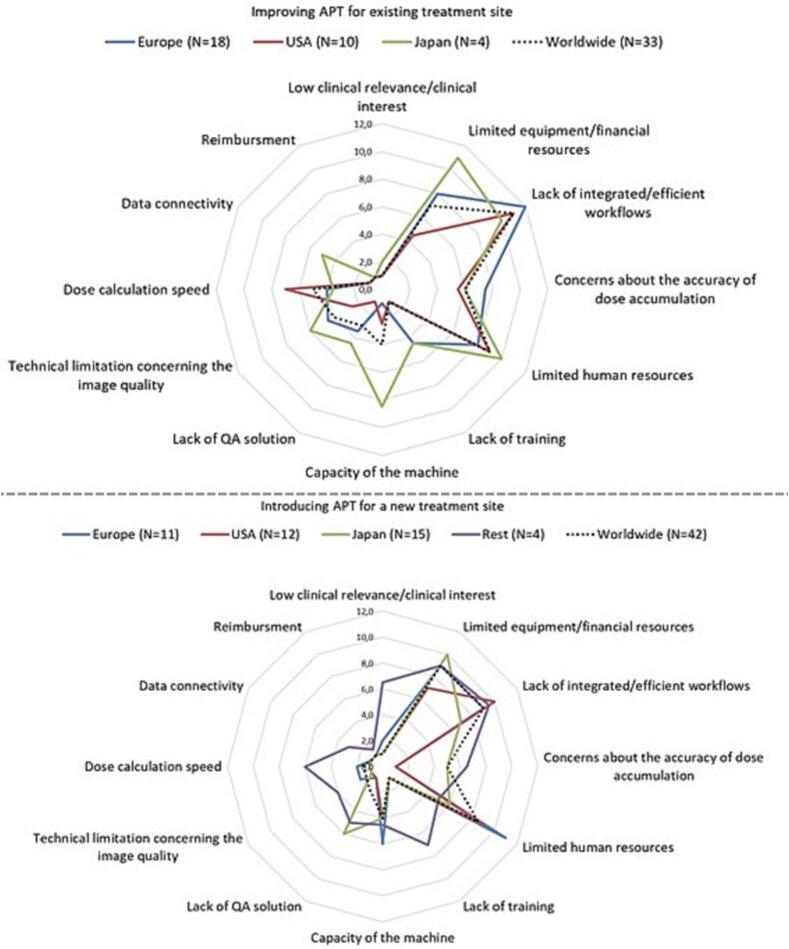
Median scoring of the barriers in improving APT for a site already treated with APT (upper figure) and in implementation of APT for a new treatment site (lower figure). The barriers were scored from 1 to 12 with 12 corresponding to the most important barrier. Each value could be used only once. The responders could leave the score empty for the barriers they did not consider relevant. In that case, lowest score (1) was assigned to that barrier. The number of users (upper figure) or responders (lower figure) is provided in the brackets. For the region “Rest” only one centre scored barriers for improving APT and low clinical relevance was the main barrier for them.

The barriers to change the APT technique for tumour sites already treated with APT were scored by 35 users and the barriers to implement APT for a new tumour site were scored by 42 responders (Fig. 4). Lack of integrated workflows, limited human or equipment and financial resources were identified as the main barriers in both cases. Clinical relevance/interest had the lowest score or was considered not relevant.

**Fig. 5:**
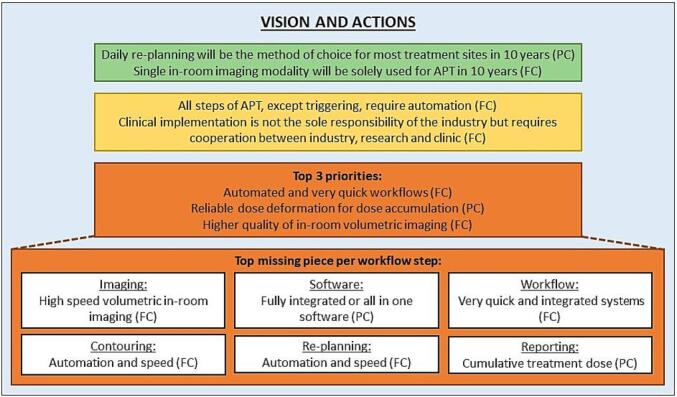
A summary of the most important conclusion from 3 round DELHI consensus analysis among 11 experts. The statements reflect results of POP ART PT survey as well as personal opinions of the individual experts. FC refers to full consensus, i.e. all experts agree. PC is partial consensus when one expert had a different opinion. Green box summarizes the vision, yellow boxes general requirements and orange boxes priorities. (For interpretation of the references to colour in this figure legend, the reader is referred to the web version of this article.)

Fig. 5 summarizes the most important outcomes from the Delphi analysis (details in Suppl. C). The Delphi analysis among the authors focused mainly on daily re-planning and its role in the future with a vision at 10-years. The outcome confirmed that the lack of integrated and efficient workflows is the largest obstruction in the implementation of daily re-planning and provided more insights on what the top priorities in implementation should be, outlined in Fig. 5.

## 4 Discussion

We report on the status of APT in clinical practice for 68 clinically operational PT centres from 17 countries worldwide, in three main regions. Eighty-four percent of clinical responders performed APT for at least one treatment site, most commonly for head and neck, with offline ad-hoc APT as the most common strategy. Furthermore, based on the responders’ wishes and barriers, we performed a Delphi consensus analysis to derive a vision for APT and provide recommendations on what should be implemented with the highest priority. Daily re-planning was determined to be the method of choice at the 10-year horizon, requiring automated and quick workflows.

The percentage of APT users was higher than for photon therapy (84% vs 61%) [28]. This can be attributed to the higher sensitivity of PT treatment to uncertainties, especially in range [1], [31], [32]. On the other hand, only two PT centres used plan-library (4% of the users) and none daily re-planning whereas, in photon therapy, 23% of users had implemented online approaches (17% plan-library, 6% daily re-planning; all with MR-linacs except one using CT-on-rail for cervix cancer) [28]. While clinical implementation of online adaptation in photon therapy, especially daily re-planning, has thrived in recent years [9], [33], research efforts for online APT have not yet made this translational breakthrough due to several factors. Firstly, PT is still relatively “young” compared to photon therapy and most of the past developments focused on the improvements of the delivery technology [34], [35]. In fact, 46% of responders had less than 5 years of experience (Suppl. Fig. B.1). New centres likely started with non-APT indications like brain tumours, implementing complicated APT sites at a later stage. Secondly, the tools required for the use of 3D in-room imaging for daily re-planning are not yet available for PT [32]. Nevertheless, validation of possible future clinical workflows is ongoing [36].

The main imaging modality was CT (77% of the users), either in separate room or in-room. As cone-beam CT image quality limits the accuracy of dose calculation, it was not yet directly used for plan re-optimization [37]. Because PT has a high sensitivity to stopping power uncertainties, the use of cone-beam CT-based treatment planning requires additional corrections for PT compared to photon therapy [38], [39]. Alternatively, in-room CTs providing planning CT quality could be used in APT [40]. Even though some users had in-room CT available, none of them used it for online APT potentially due to workflow complexity leading to longer treatment room occupancy, high demand on human resources and comprehensive QA requirements. In contrast to photon therapy, MRI-guided PT is currently not available but under active development with a first prototype already built [41]. It is still an open research question if a suitable synthetic CT based on MR images for dose calculation can be generated [41], [42], [43], [44]. In the Delphi analysis, there was no consensus on the future role of MRI guided particle therapy.

Regarding QA of the adapted treatment plans, APT relied mainly on pre-treatment measurements, similarly to photon therapy [28]. A clear difference was the relatively high proportion of in-house software use or mentions of inadequate commercial solutions compared to photon therapy. Nevertheless, lack of QA solution was not scored as a high burden. Ongoing research focuses on alternative solutions to replace measurements with the same level of confidence. Log-file based dose calculations with independent dose engine as an in-silico patient specific QA may provide results consistent with measurements [45].

Most of the users would like to improve the APT workflow for a treatment site already treated with APT or implement APT for a new treatment site. Interest was highest in the US. In Japan, APT is well-established and wishes for changes and further implementation were low. In general, the barriers were scored similarly to the POP-ART RT survey [28], although, lack of integrated workflows came ahead of human/material resources in PT. There were small variations among the regions: The “rest” of the world scored *lack of training* higher than other regions for the introduction of APT for a new treatment site, but this was based only on four responders.

The Delphi analysis reached a consensus on daily re-planning being the method of choice at the 10-year horizon (PC) and being performed with a single in-room imaging modality (FC). There was no consensus on whether offline adaptation would still be performed once online APT is used clinically, as in 10 years, adaption addressing tumour shrinkage or biological response may be possible [32], starting in an offline manner.

Although there was full consensus among the authors on the need for automation to bring online APT to broad-scale clinical reality, triggering of the adaptation cannot be standardized yet as we do not have enough experience. Artificial Intelligence was seen as an asset for automation, especially in auto-segmentation where it can also help reduce inter/intra-observer variability.

No consensus was reached among the authors on the best method for patient-specific QA in online APT. Many options were listed starting from measurements and/or independent dose calculations, sanity checks, log-file dose calculation, independent dose calculation combined with measurements only from time to time to verify independent dose calculations, comparison to non-adapted plans and evaluation of the order of magnitude changes in spots weights, however, opinions on what the best approach is differ largely. Further developments, investigations and discussions in the proton therapy community are needed to answer this question.

An important consensus recommendation was that clinical implementation requires cooperation between industry, research and clinic with the top three priorities identified as: (1) Automated and very quick systems that do not degrade the throughput and reduce human interaction. This has to be addressed by research (finding the most reliable method), industry (to provide the systems) and clinics (to evaluate the clinical applicability). These systems should be able to communicate with record and verify systems. (2) Reliable deformable registration for dose accumulation and tools to estimate dose accumulation uncertainty. It should also be a combined effort of industry and research. (3) Higher quality of in-room volumetric imaging with daily synthetic CTs (from CBCTs) solution. Industry is needed for developing high quality imaging system and research for dose calculation on CBCT.

Our study has several limitations. Data collection relied on the centres willing to respond to the survey. Some answers were estimations from the person answering the survey. Another person from the same institution might have provided different answers. The survey was designed with the aim to be answered within 15 min to encourage participation and provide a helicopter view of status and needs; therefore, other potentially important questions might have been omitted. The responders had the opportunity to provide free-form comments. These answers are clearly marked in results as it could create a bias; other centres might do/think the same but did not provide a comment. The authors participating in Delphi have different level of involvement in clinical and research tasks as well as proton therapy experience.

In conclusion, 70 PT centres from 17 countries worldwide participated in the survey and provided an overview of the APT implementation status as well as the wishes and barriers to further use and implementation. Offline APT is currently the state-of-art with daily re-planning seen as the method of choice at the 10-year horizon. Even though PT treatments are more sensitive to interfractional changes compared to photon therapy, the implementation of online daily APT is currently still limited with only two centres using plan-library. Joint efforts between industry research and clinics are needed to translate innovations into efficient and clinically feasible workflows for broad-scale implementation.

## Declaration of Competing Interest

The authors declare that they have no known competing financial interests or personal relationships that could have appeared to influence the work reported in this paper.
