# Supplementary material for: A survey of practice patterns for adaptive particle therapy for interfractional changes
Source: Phys Imaging Radiat Oncol. 2023 Apr 28;26:100442. doi: 10.1016/j.phro.2023.100442 (PMC10183663; doi:10.1016/j.phro.2023.100442)
Supplement: Supplementary data 1 [file mmc1.docx]

# Supplementary Material A

Full survey for the part II of the POP ART PT

## General questions (same for part I and part II)

**Q1:** What is the name of your institution?

**Q2:** In which country are you situated?

**Q3:** Your institution is ... (please tick all that apply):

- Private
- Public
- Academic

**Q4:** For how many years has your particle centre been in clinical operation?

**Q5:** How many patients is your institution currently treating with particle therapy per year (approximately)?

**Q6:** How many patients does your institution plan to treat with particle therapy in full ramp-up per year (approximately)?

**Q7:** How many clinical treatment rooms does your particle therapy facility have (excluding eye-lines)?

**Q8:** How many clinical treatment rooms are currently in operation in your particle therapy facility (excluding eye-lines)?

**Q9:** Which treatment delivery machine do you have?

- Varian
- IBA PPlus
- IBA Pone
- Mevion
- Hitachi
- Other (please specify)

**Q10:** Please provide an email address in case we need to contact you for clarification (voluntary)

## Adaptive particle therapy (APT) for interfractional anatomical changes using multiple plans

**Q11:** Are you doing APT for any tumour site (even if just for a subgroup of patients) in your particle therapy facility?

- Yes, continue to site-specific questions.
- No, skip to plans and wish list section.

**Q12:** For which tumour sites are you doing APT (even if just for a patient subgroup)?

- Bladder
- Cervix
- Rectum
- Prostate
- Head and Neck
- Lung
- Other (please specify)

**Q13:** What type of adaptation are you using for this site?

| Site | Offline  Ad-hoc replanning | Offline replanning with protocol | Plan Library approach | Daily replanning | N/a |
| --- | --- | --- | --- | --- | --- |
| bLADDER | 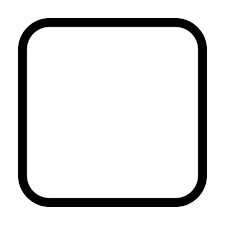 | 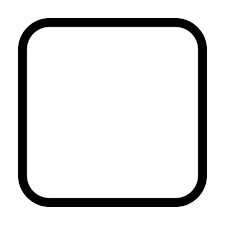 | 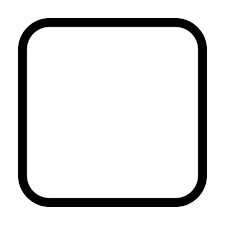 | 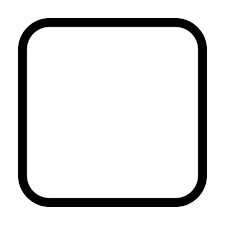 | 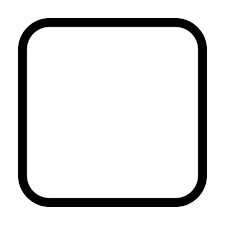 |
| cERVIX | 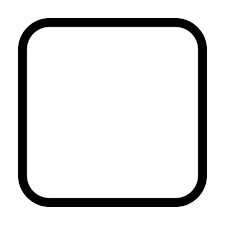 | 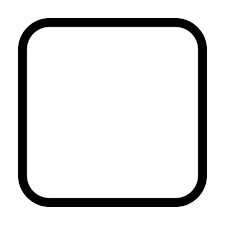 | 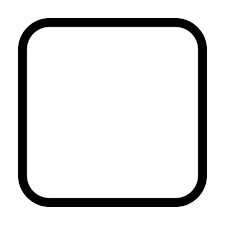 | 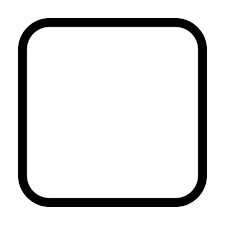 | 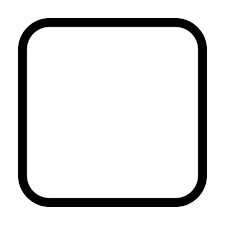 |
| rECTUM | 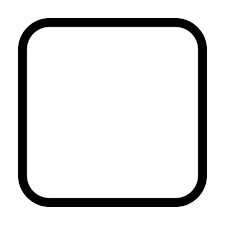 | 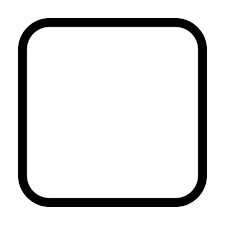 | 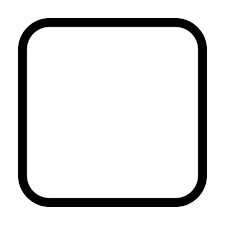 | 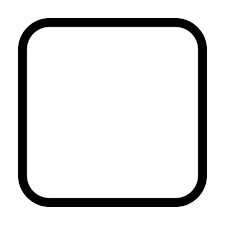 | 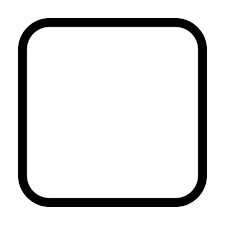 |
| PROSTATE | 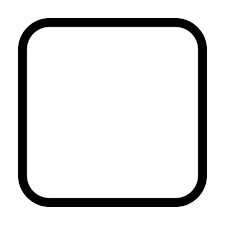 | 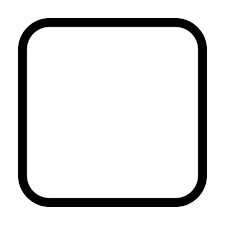 | 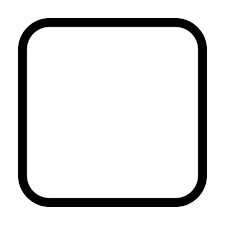 | 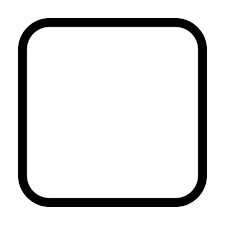 | 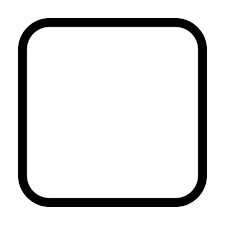 |
| HEAD AND NECK | 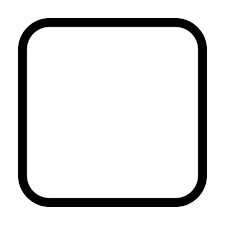 | 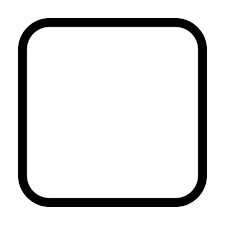 | 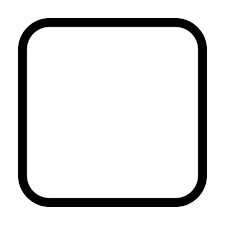 | 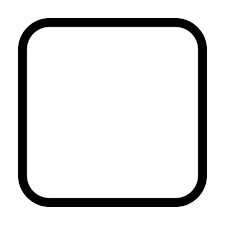 | 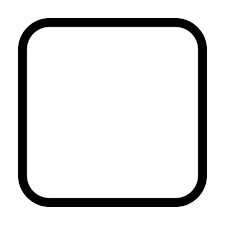 |
| LUNG | 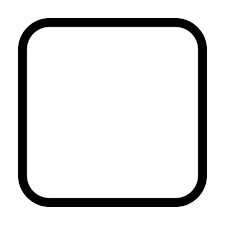 | 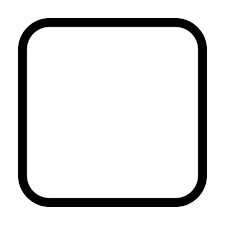 | 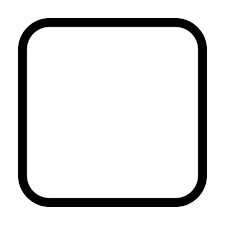 | 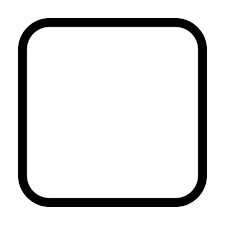 | 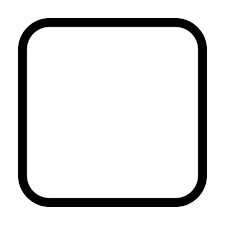 |
| OTHER* | 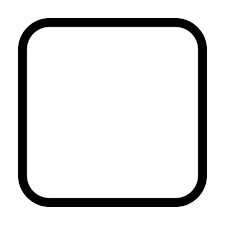 | 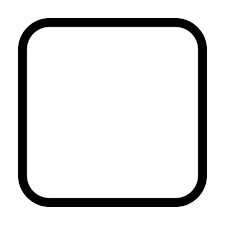 | 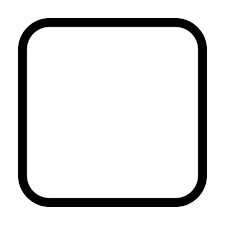 | 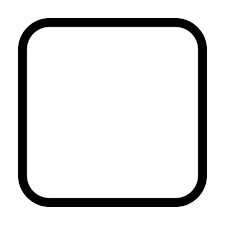 | 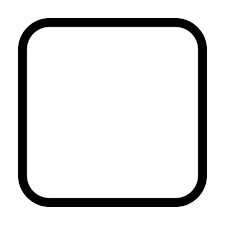 |

** Other (please specify)*

**Q14:** If off-line re-planning (ad-hoc or protocolled): What percentage of patients approximately is treated with more than one plan?

| Site | < 5% | 5-25% | 25-50% | 50-75% | 75-100% |
| --- | --- | --- | --- | --- | --- |
| bLADDER | 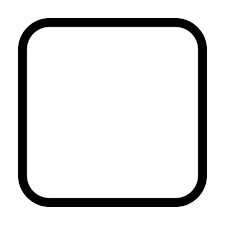 | 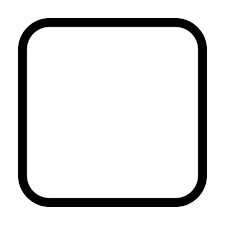 | 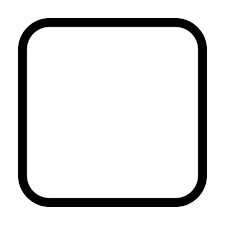 | 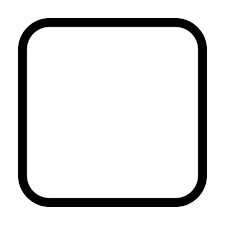 | 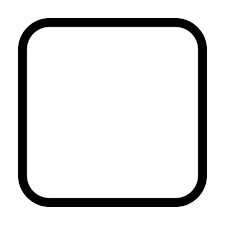 |
| cERVIX | 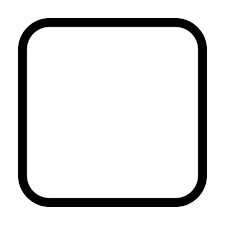 | 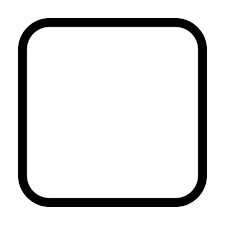 | 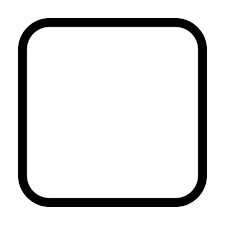 | 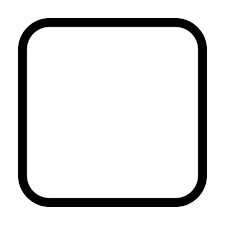 | 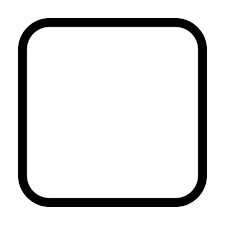 |
| rECTUM | 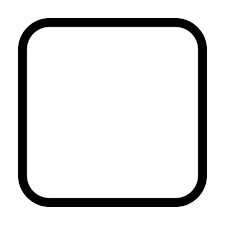 | 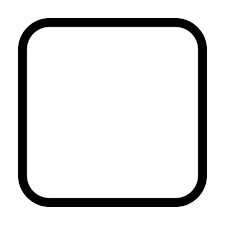 | 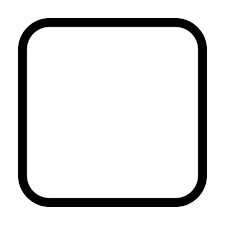 | 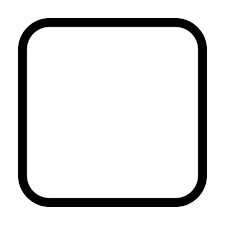 | 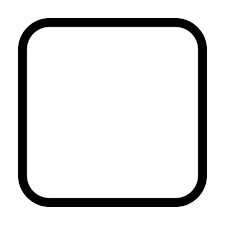 |
| PROSTATE | 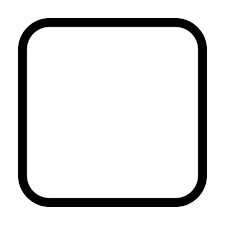 | 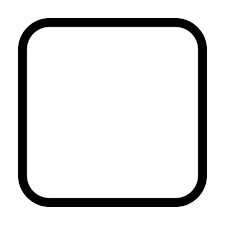 | 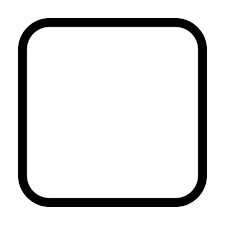 | 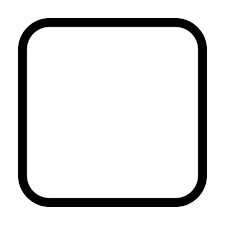 | 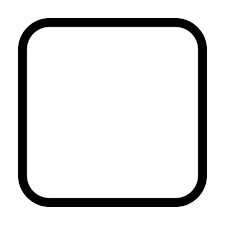 |
| HEAD AND NECK | 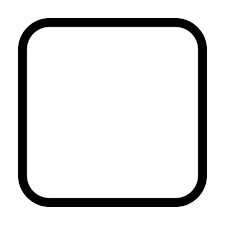 | 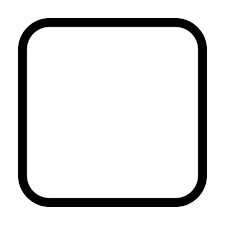 | 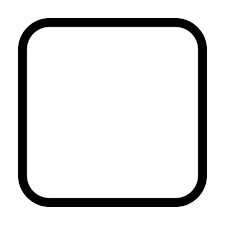 | 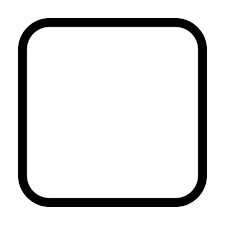 | 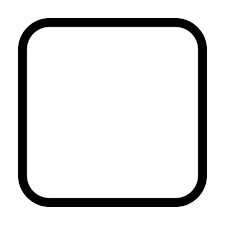 |
| LUNG | 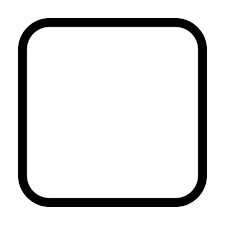 | 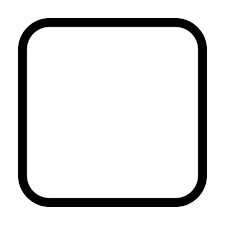 | 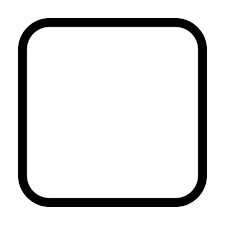 | 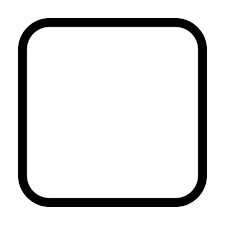 | 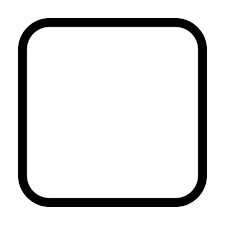 |
| OTHER* | 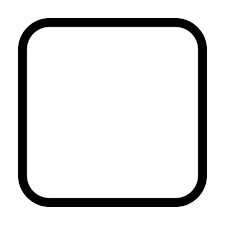 | 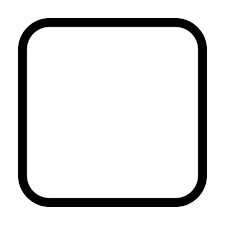 | 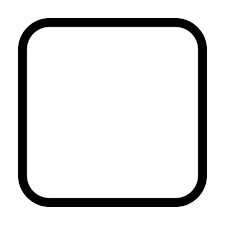 | 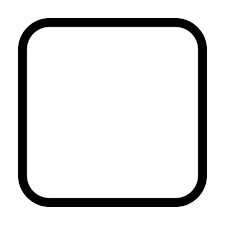 | 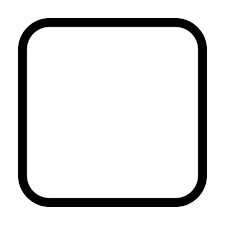 |

** Other (please specify)*

**Q15:** Why are you doing APT (multiple answers possible)?

| Site | Target dose considerations | oar dose considerations | N/a |
| --- | --- | --- | --- |
| bLADDER | 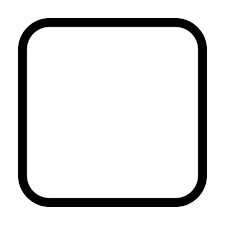 | 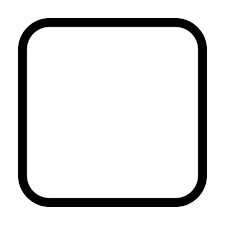 | 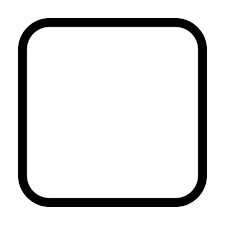 |
| cERVIX | 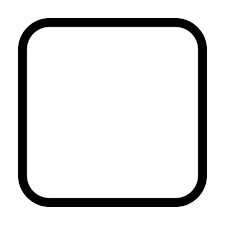 | 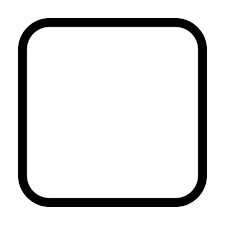 | 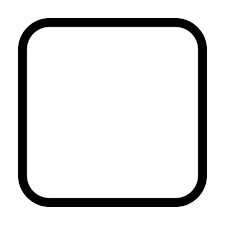 |
| rECTUM | 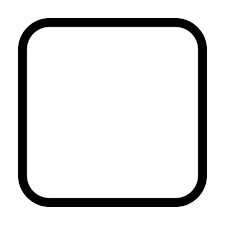 | 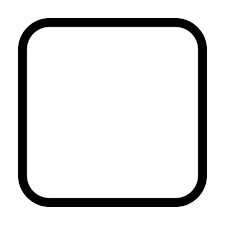 | 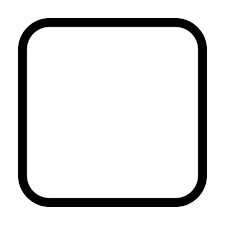 |
| PROSTATE | 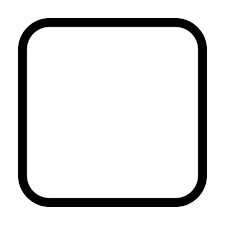 | 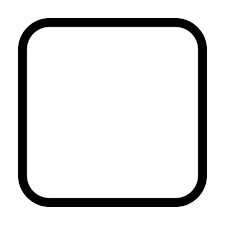 | 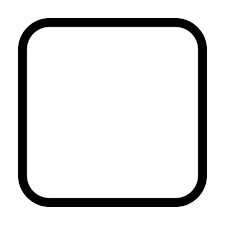 |
| HEAD AND NECK | 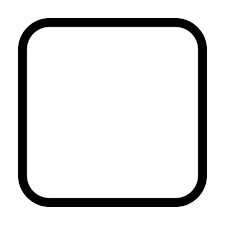 | 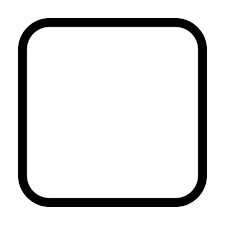 | 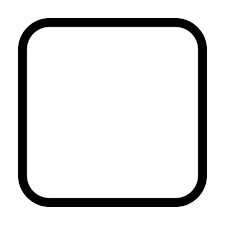 |
| LUNG | 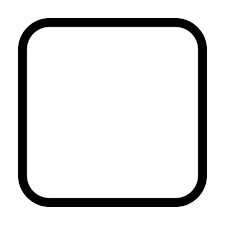 | 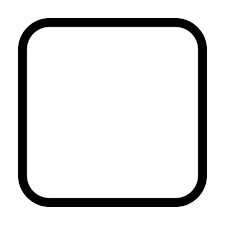 | 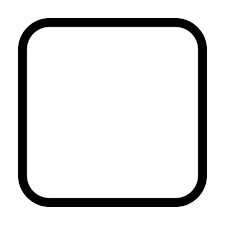 |
| OTHER* | 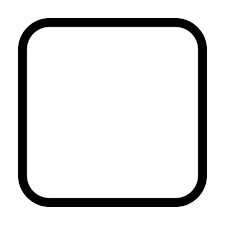 | 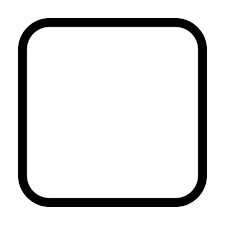 | 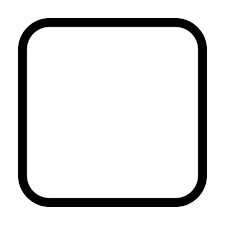 |

** Other considerations (please specify which and for which site)*

**Q16:** Which repeated imaging are you acquiring to decide on adaptation? Please also indicate if it is in room imaging and the frequency (Here we are NOT considering extra imaging to make the new plan once the decision to adapt has already been taken).

| Site | CBCT | kV | ct | mr | surface | online | offline |
| --- | --- | --- | --- | --- | --- | --- | --- |
| bLADDER | 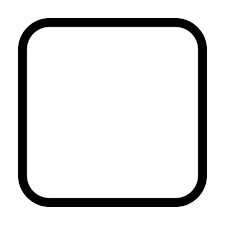 | 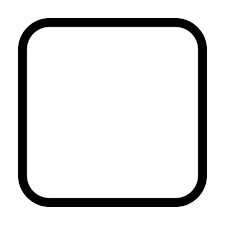 | 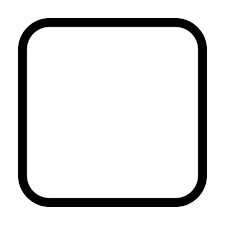 | 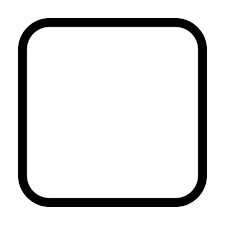 | 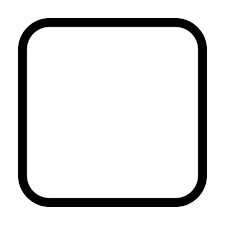 | 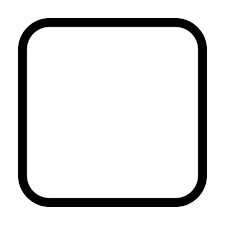 | 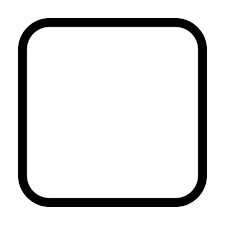 |
| cERVIX | 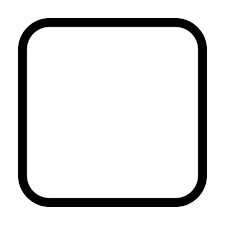 | 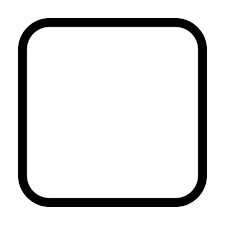 |  |  |  |  |  |
| rECTUM |  |  |  |  |  |  |  |
| PROSTATE |  |  |  |  |  |  |  |
| HEAD AND NECK |  |  |  |  |  |  |  |
| LUNG |  |  |  |  |  |  |  |
| OTHER* |  |  |  |  |  |  |  |

** Other (please specify)*

| Site | at each fraction | at specific fraction | ad-hoc |
| --- | --- | --- | --- |
| bLADDER |  |  |  |
| cERVIX |  |  |  |
| rECTUM |  |  |  |
| PROSTATE |  |  |  |
| HEAD AND NECK |  |  |  |
| LUNG |  |  |  |
| OTHER* |  |  |  |

** Other (please specify)*

**Q17:** Which information is triggering the adaptation (multiple answers possible)?

| Site | visual inspection of repeated images | dosimentric evaluation on the repeated images | treatment related anatomical changes | other patient parameters | N/a |
| --- | --- | --- | --- | --- | --- |
| bLADDER |  |  |  |  |  |
| cERVIX |  |  |  |  |  |
| rECTUM |  |  |  |  |  |
| PROSTATE |  |  |  |  |  |
| HEAD AND NECK |  |  |  |  |  |
| LUNG |  |  |  |  |  |
| OTHER* |  |  |  |  |  |

** Other triggers (please specify which and for which site)*

**Q18:** For which parts of the adaptive workflow do you use what kind of software? Also, indicate if these tasks are performed automatically, semi-automatically or manually.

| Site | in-house | commercial | open source | not in workflow | N/a |
| --- | --- | --- | --- | --- | --- |
| image registration |  |  |  |  |  |
| oar contouring |  |  |  |  |  |
| target contouring |  |  |  |  |  |
| plan recalculation |  |  |  |  |  |
| plan evaluation |  |  |  |  |  |
| adaptation triggering |  |  |  |  |  |
| plan reoptimization |  |  |  |  |  |
| plan qa |  |  |  |  |  |

| Site | Automatically | Semi-automatically | manually | N/a |  |
| --- | --- | --- | --- | --- | --- |
| image registration |  |  |  |  |  |
| oar contouring |  |  |  |  |  |
| target contouring |  |  |  |  |  |
| plan recalculation |  |  |  |  |  |
| plan evaluation |  |  |  |  |  |
| adaptation triggering |  |  |  |  |  |
| plan reoptimization |  |  |  |  |  |
| plan qa |  |  |  |  |  |

Additional comments:

**Q19:** If "in-house" for the previous question: Why not a commercial/open source solution?

| Site | Too expensive | Not good enough | does not offer needed functionalities | not commissioned |
| --- | --- | --- | --- | --- |
| image registration |  |  |  |  |
| oar contouring |  |  |  |  |
| target contouring |  |  |  |  |
| plan recalculation |  |  |  |  |
| plan evaluation |  |  |  |  |
| adaptation triggering |  |  |  |  |
| plan reoptimization |  |  |  |  |
| plan qa |  |  |  |  |

| Site | Lack of connectivity between software | No commercial or open source software available | N/A |
| --- | --- | --- | --- |
| image registration |  |  |  |
| oar contouring |  |  |  |
| target contouring |  |  |  |
| plan recalculation |  |  |  |
| plan evaluation |  |  |  |
| adaptation triggering |  |  |  |
| plan reoptimization |  |  |  |
| plan qa |  |  |  |

Additional comments:

**Q20:** What QA is performed on the new adapted plan(s)? Also indicate if it is the same or different from the QA of the original plan.

| Site | none | pre-treatment phantom measurements | post-treatment phantom measurements | secondary dose calculation | log file analysis |
| --- | --- | --- | --- | --- | --- |
| bLADDER |  |  |  |  |  |
| cERVIX |  |  |  |  |  |
| rECTUM |  |  |  |  |  |
| PROSTATE |  |  |  |  |  |
| HEAD AND NECK |  |  |  |  |  |
| LUNG |  |  |  |  |  |
| OTHER |  |  |  |  |  |

| Site | same as for original qa plan | different from the original QA plan | N/a |
| --- | --- | --- | --- |
| bLADDER |  |  |  |
| cERVIX |  |  |  |
| rECTUM |  |  |  |
| PROSTATE |  |  |  |
| HEAD AND NECK |  |  |  |
| LUNG |  |  |  |
| OTHER |  |  |  |

If the QA for the adapted plan is different from the QA for the original plan specify briefly the difference:

**Q21:** Which tool do you use to manage and document plan adaptation

| Site | none | record & verify software | spreadsheet (e.g. excel) | TPS | other (specify) | N/A |
| --- | --- | --- | --- | --- | --- | --- |
| bLADDER |  |  |  |  |  |  |
| cERVIX |  |  |  |  |  |  |
| rECTUM |  |  |  |  |  |  |
| PROSTATE |  |  |  |  |  |  |
| HEAD AND NECK |  |  |  |  |  |  |
| LUNG |  |  |  |  |  |  |
| OTHER |  |  |  |  |  |  |

## Plans and wishes: Adaptive particle therapy (APT)

**Q22:** Do you have plans to expand the use or change/improve your APT technique for an existing tumour site in the next 2 years?

- Yes
- No

**Q23:** For which tumour site(s) do you wish to expand the use or change/improve your APT technique in priority?

**Q24:** What are the main barriers/challenges to wider use of PT? (Rank in order of importance where 1 is the greatest challenge, leave the choices that are considered not relevant unmarked):

- Low clinical relevance/clinical interest
- Limited equipment/financial resources
- Lack of integrated/efficient workflows
- Concerns about the accuracy of dose accumulation
- Limited human resources
- Lack of training
- Capacity of the machine
- Lack of QA solution
- Technical limitation concerning the image quality
- Dose calculation speed
- Data connectivity
- Reimbursement

**Q25:** Any other main barrier not specified above?

**Q26:** Do you have wishes to implement APT for anew tumour site?

- Yes, we want to implement APT for a new tumour site and we have plans for implementation in the next 2 years.
- Yes, but we have no plans to implement it.
- No, we have not wish to implement adaptive radiotherapy for a new tumour site.

**Q27:** For which tumour site(s) do you plan or would you like to implement APT in priority?

**Q28:** What are the main barriers/challenges to implement APT for a new indication? (Rank in order of importance where 1 is the greatest challenge, leave the choices that are considered not relevant unmarked)

**Q29:** Any other main barrier not specified above?

# Supplementary Material B

An overview of the general statistics of responding centres as well as some additional results are provided here:

- Information on response rate: Table B.1
- Academic status: Table B.2
- Machine vendors: Table B.3
- Years of operation: Figure B.1
- Number of patients: Figure B.2
- Percentage of patients having more than one plan per treatment site: Figure B.3
- Imaging devices: Figure B.4
- Location of imaging devices: Figure B.5
- Frequency of imaging: Figure B.6
- Number of APT treatment sites: Table B.4
- An overview of treated indications: Table B.5
- Ratios of centres per region planning to improve the APT: Figure B.7
- Scoring of barriers in implementation per region: Figure B.8

*Table B.1: An overview of the numbers of responding centres and information on the clinical operation status.*

*Table B.2: An overview of the academic status of the responding particle therapy centres.*

| Region | Academic | Non-academic |
| --- | --- | --- |
| Europe | 10 | 13 |
| USA | 13 | 7 |
| Japan | 5 | 17 |
| Rest | 2 | 3 |
| **World** | **30** | **40** |

*Table B.3: An overview of the vendors in the responding particle therapy centres (N=70). *Combination=two different systems were used in a centre.*

*Figure B.1: An overview of the years of experience of the responding particle therapy centres. The upper graph describes the experience of all the centres worldwide (N=70) and the lower plots describe the experience per region.*

*Figure B.2: An overview of the number of treated patients per year at the operational responding particle therapy centres. The upper graph left describes the numbers of all the centres worldwide (N=64) and right the amount of the centres at a full patient capacity. The lower plots describe the patient numbers per region.*

*Figure B.3: An overview of the percentage of patients having more than one plan per treatment site, i.e original plan and at least one adaptation of the original plan. The percentage of users is related to the number of users (N) per treatment site.*

*Figure B.4: An overview of imaging devices used for certain steps of APT. The category “combination” indicates if more than one imaging device is used by a certain centre. The percentage of users is related to the number of users (N) per treatment site.*

*Figure B.5: An overview of location of imaging devices. The percentage of users is related to the number of users (N) per treatment site. Due to the structure of the survey it was not possible to conclude which device are located in-room and which in separate room as well as which type of imaging is used for what type of workflow.*

*Figure B.6: An overview of frequency of imaging for APT. The percentage of users is related to the number of users (N) per treatment site.*

*Table B.4: An overview of the number of indications treated with APT in different regions of 57 users. The N indicates number of APT users per region.*

*Table B.5: An overview of the other indications treated with APT.*

*Figure B.7: Percentage of centres within different regions that plan, wish or do not wish to change their APT workflow (improve APT) or implement APT for a new treatment site (new APT). The evaluation is based on the answers of 54 APT users and 16 non-users including not operational responders (squared pattern).*

*Figure B.8: List of treatment site for which the centres wanted to improve APT or newly introduce. All APT sites refer to all the tumour sites currently treated with APT at a given centre.*

# Supplementary Material C

To reach a consensus on what are the most important developments, a survey was conducted among all authors of the manuscript, experts in the field of adaptive radiotherapy using the Delphi method [30]. The expertise of each author is summarized in a Table C.1. The moderator of the survey also participated in answering the Delphi questions, however always prior to reading answers of the others.

A three-round questionnaire was developed following the scheme in Figure C.1:

- First round: A concept questionnaire was created by four authors (PT, YZ, AK, JB). All authors answered the questions and sent their answers to the moderator of the analysis (PT). The authors that were not involved in the drafting of the questionnaire had an opportunity to add missing questions or suggest removal of irrelevant questions (no questions were removed, questions 2 and 3 were added).
- Second round: The moderator summarised the answers, added new questions and redistributed the questionnaire with all answers in an anonymized form to generate controlled opinion feedback. Authors could revise their answers and comment on the answers from others. The moderator again collected all the answers.
- Third round: Based on the answers from second round, the moderator defined the final statements with only agree/disagree answer possible and distributed the statements. A summary of the answers from the second round in anonymized form was provided together with the statements and it was still possible to comment on the answers of the others.

Full consensus (FC), partial consensus (PC) or no consensus (NC) were reached when all experts have agreed on an answer, only one expert had a different opinion or more than one expert had a different opinion, respectively.

The overview of the questions from the final round together with all the answers is presented in Table C.2. Sometimes more than one answer for a question was possible. Not all the participants have answered all the questions. Every participant had a possibility to comment each question to elaborate on their answer.

*Table C.1: Experience level of participants of Dephi consensus analysis (status from March 2023).*

*Figure C.1: A structure of Delphi consensus process based on 11 participants.*

*Table C.2: An overview of questions and answers of the third round of DELHI analysis. The numbers (x/n) in brackets indicate x= number of answers and n= number of participants. FC = full consensus, PC = partial consensus and NC = no consensus. The answers highlighted in bold correspond to FC.*
